# HTLV-1 Tax-activated NF-κB is involved in both cell growth and death

**DOI:** 10.1186/1742-4690-11-S1-P93

**Published:** 2014-01-07

**Authors:** Mariko Mizuguchi, Masaya Higuchi, Masahiro Fujii, Masataka Nakamura

**Affiliations:** 1 Human Gene Sciences Center, Tokyo Medical and Dental University, Tokyo, Japan; 2 Division of Virology, Niigata University Graduate School of Medical and Dental Sciences, Niigata, Japan

## 

HTLV-1 Tax (Tax1) dysregulates the expression of cellular genes through transcriptional factors such as NF-kB, CREB and AP-1, leading to cell proliferation and immortalization. Conversely, Tax1 is reported to induce growth arrest and apoptosis. The molecular mechanism underlying growth arrest and cell death by Tax1 remains to be elucidated. The interleukin 2 (IL-2)-dependent human cell line Kit 225, in which the resting phase of cell growth is induced by deprivation of IL-2, was used to examine effects of Tax1 in growing and resting Kit 225 cells. Introduction of Tax1 reduced cell growth in growing cells, while Tax1 enhanced cell activity in resting cells. Tax1 activated the cell cycle transcription factor E2F and also increased the expression of cell cycle regulatory genes such as CDK2, CDK4 and cyclin D2 in resting cells. Tax1-induced reduction of cell growth was associated with apoptosis. No reduction in cell growth was observed in growing cells after introduction of Tax2 coded by HTLV-2, which has not been shown to link to malignant diseases. Tax1-induced cell growth reduction and apoptosis were demolished by siRNA against the NF-kB subunit RelA. In contrast, resting Kit 225 cells reduced cell activity by decreasing RelA upon Tax1 introduction. These results indicate that Tax1-activated RelA acts on both cell growth and cell death in a cell cycle-dependent manner. Functional differences between Tax1 and Tax2 may influence HTLV-1 pathogenesis.

